# Supplementary material for: OpenMM 7: Rapid development of high performance algorithms for molecular dynamics
Source: PLoS Comput Biol. 2017 Jul 26;13(7):e1005659. doi: 10.1371/journal.pcbi.1005659 (PMC5549999; doi:10.1371/journal.pcbi.1005659)
Supplement: S1 Source Code — (ZIP) [file pcbi.1005659.s002.zip › OpenMM-7.0.1-Source/docs/api-python/generated/simtk.openmm.app.internal.charmm.exceptions.CharmmPSFWarning.html]

CharmmPSFWarning — OpenMM 7.0.1.dev-5e86c4f documentation


# CharmmPSFWarning¶

*class* `simtk.openmm.app.internal.charmm.exceptions.``CharmmPSFWarning`¶
:   For non-fatal PSF parsing issues

    `__init__`()¶
    :   x.\_\_init\_\_(...) initializes x; see help(type(x)) for signature

    `__delattr__`¶
    :   x.\_\_delattr\_\_(‘name’) <==> del x.name

    `__format__`()¶
    :   default object formatter

    `__getattribute__`¶
    :   x.\_\_getattribute\_\_(‘name’) <==> x.name

    `__getitem__`¶
    :   x.\_\_getitem\_\_(y) <==> x[y]

    `__getslice__`¶
    :   x.\_\_getslice\_\_(i, j) <==> x[i:j]

        Use of negative indices is not supported.

    `__hash__`¶

    `__reduce_ex__`()¶
    :   helper for pickle

    `__repr__`¶

    `__setattr__`¶
    :   x.\_\_setattr\_\_(‘name’, value) <==> x.name = value

    `__sizeof__`() → int¶
    :   size of object in memory, in bytes

    `__str__`¶

High performance molecular simulation on GPUs


### Navigation

- Application Layer
  - Loaders and Setup
  - Representation and Manipulation
  - Simulation
  - Reporting Output
  - Extras
- Library Layer

©2015, Stanford University and the Authors.
|
Powered by Sphinx 1.3.1
& Alabaster 0.7.7
|
Page source
